# Quantification of Carbonic Contamination of Fused Silica Surfaces at Different Stages of Classical Optics Manufacturing

**DOI:** 10.3390/ma14071620

**Published:** 2021-03-26

**Authors:** Robert Köhler, Domenico Hellrung, Daniel Tasche, Christoph Gerhard

**Affiliations:** 1 Faculty of Engineering and Health, HAWK University of Applied Sciences and Arts, Von-Ossietzky-Str. 99/100, 37085 Göttingen, Germany; robert.koehler@hawk.de (R.K.); domenico.hellrung@stud.hawk.de (D.H.); daniel.tasche2@hawk.de (D.T.); 2 Faculty of Natural and Materials Science, Clausthal University of Technology, Robert-Koch-Straße 42, 38678 Clausthal-Zellerfeld, Germany

**Keywords:** glasses, surface chemistry, surface analysis, optics manufacturing, carbonic contamination, roughness

## Abstract

The chemical composition of ground and polished fused silica glass surfaces plays a decisive role in different applications of optics. In particular, a high level of carbon impurities is often undesirable for further processing and especially for gluing or cementing where adhesion failure may be attributed to carbonic surface-adherent contaminants. In this study, the surface carbon content at different stages of classical optics manufacturing was thus investigated. Two different standard processes—grinding and lapping with two final polishing processes using both polyurethane and pitch pads—were considered. After each process step, the chemical composition and roughness of the surface were analysed using X-ray photoelectron spectroscopy and atomic force microscopy. An obvious correlation between surface roughness and effective surface area, respectively, and the proportion of carbon contamination was observed. The lowest carbon contamination was found in case of lapped and pitch polished surfaces.

## 1. Introduction

In the course of classical optics manufacturing, glass surfaces are in permanent contact with abrasive tools and operating materials. Most of these media contain carbon, hydrocarbons or carbonaceous compounds. For instance, grinding and lapping is usually performed with abrasives made of diamond or silicon carbide. Moreover, either polyurethane foil—consisting of carbamate groups—or pitch—consisting of hydrocarbons—is used as polishing pad material [1]. Finally, tap water widely used in optics manufacturing usually contains a considerable amount of carbon [2,3] given by carbonic acid, salts as well as dissolved and undissolved total organic carbon (TOC). In some manufacturing steps or special cases, further carbonaceous substances are added to the water, for example, mineral oil for mixing the cooling lubricant used for cutting and bound abrasive grinding or defoamers that are sometimes applied during polishing.

It is obvious that to a certain extent, the glass surface interacts with the used tools and operating materials. Quite an amount of published work focusses on the investigation of impurities induced by the polishing process and the used polishing agents, namely cerium, iron, aluminium, lanthanum, etc. [4,5,6,7,8,9,10]. However, manufacturing-induced contamination of glass surfaces by carbon is less considered in literature, even though the presence and disturbing effect of carbonaceous contamination layers is a well-known phenomenon [11,12]. It is normally attributed to pervasive hydrocarbon contamination overlayers [13] induced by the adsorption of gases from the ambient air [14].

The analysis of the origin and grade of carbon contamination in the course of classical optics manufacturing is of specific interest for the production of high performance optics where the lowest contaminants may cause a failure in functionality. This includes several aspects or effects: (i) In the case of laser optics, the laser-induced damage threshold (LIDT) of glass surfaces might be significantly lowered due to absorption of incoming laser light at carbonic impurities [8,15]. Such impurities are embedded either in micro cracks and subsurface damages or within the silica gel layer that is formed in the course of polishing [5]. (ii) The presence of carbon or carbonic groups may, moreover, cause the degradation of surface adhesion. However, high adhesion is required for quite different approaches where, for example, lens doublets or prism pairs are produced. This applies to optical contact bonding [16] via adhesion due to intermolecular forces [17,18], classical cementing where polished surfaces are connected using fine cements [1], and gluing of ground or lapped lens borders and rims in holders, mounts and tubes [1]. (iii) Surface-adherent C=C bonds are known to be hygroscopic [19] and thus act as functional groups for improved adhesion of water. The water attracted by the presence of such carbon compounds may give rise to several glass defects. This includes the so-called glass corrosion [20], where a greyish haze is formed at the polished glass surface due to a water-induced hydrolytic scission of the glass network.

It thus turns out that knowledge about the presence and origin of carbon is of interest in modern optics manufacturing technology. In previous work it has been shown that the contamination of polished glass surfaces by carbon features a certain dependency on the concentration of the used polishing suspension [15]. This implies that differences in tool—glass interactions may play a notable role in the degree or amount of contamination. Against this background, carbon surface contamination of fused silica after each single production step of classical optics manufacturing was investigated in the present work. This study was performed in order to identify “critical” production steps and operating materials as well as the particular contribution of the used tools and media to carbonaceous surface contamination of the final polished surface.

## 2. Materials and Methods

### 2.1. Sample Preparation

**Figure 1:**
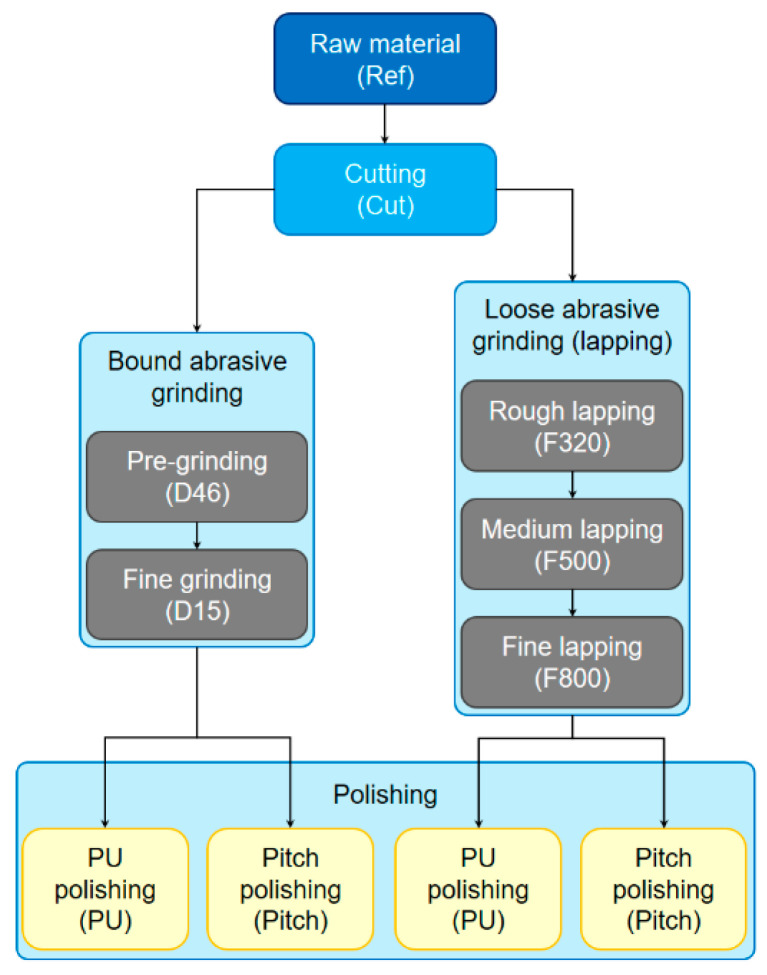
Workflow illustrating the preparation of the different investigated samples including the particular sample denomination used in this work (given in parentheses).

The sample material investigated in this work was an optical fused silica glass (Tosoh Corp., Tokyo, Japan, type Clear Silica Glass N). From the raw material, a glass block, four different series of samples were produced by different standard production processes applied in classical optics manufacturing, as shown schematically in Figure 1. The main difference between these series was the approach used for grinding on the one hand and the used polishing pad material on the other hand [1].

Initially, plane plates were cut from the fused silica block where a commercial cut-off wheel was used. The edge of this cut-off wheel was coated with diamond particles with a mean grit size of 126 µm (denomination D126, according to the FEPA standard). The particles were embedded in a bronze matrix. In the course of cutting, a mixture of water and a commercial cooling lubricant (Rhenus, Mönchengladbach, Germany, type Polinor GMC) was applied. This coolant was also used during subsequent bound abrasive grinding. Such grinding was performed with two different diamond grinding cup wheels for pre-grinding and fine grinding. The mean diamond grit sizes of these tools were 46 µm (D46) and 15 µm (D15), respectively. As for the cut-off wheel, the diamond particles were bound in a bronze matrix.

In addition to bound abrasive grinding, loose abrasive grinding, i.e., lapping, was applied after cutting to realise the second main series of samples. Here, silicon carbide was used as lapping medium. Lapping was performed in three subsequent steps where the mean grain size was successively reduced, i.e., 29 µm for rough lapping, 13 µm for medium lapping and 7 µm in the case of fine lapping. This corresponds to the lapping material designations F320, F500 and F800 according to the FEPA standard.

Finally, both bound abrasive ground and lapped samples were polished using two different approaches as represented by the applied polishing pad materials, polyurethane (PU) foil on the one hand and pitch on the other hand. In both cases, the applied polishing suspension was a commercial cerium oxide premix (Pieplow & Brand, Henstedt-Ulzburg, Germany, type CERI 3000) with a mean polishing grain size of 0.7 µm. After each single production step shown in Figure 1, the cut, ground, lapped or polished surfaces were measured as described in more detail hereafter. Before chemical analysis, the samples were cleaned with isopropyl alcohol.

### 2.2. Sample Characterisation

In order to gain information on the surface topography of the samples, both the average area roughness Sa and the root mean square area roughness Sq were detected (according to the standard ISO 4287/1-1997) after each manufacturing step. Here, an atomic force microscope (AFM) (Nanosurf GmbH, Langen, Germany, model easyScan 2) was applied where the measured area was 50 × 50 µm^2^.

Moreover, the chemical composition of the surfaces was determined by high-resolution X-ray photoelectron spectroscopy (XPS) where a scanning XPS apparatus (Ulvac-phi, Inc., Chigasaki, Japan, model PHI VersaProbe II) with a monochromatic Al-K_α_ source and a photon energy of 1486.6 eV was used. The applied X-ray source has a power of 100 W, whereby the sample surface is scanned with a beam size of 100 µm over an area of 1400 × 200 µm². High-resolution spectra were recorded with a pass energy of 46.95 eV and a step size of 0.1 eV with a constant electron take-off angle of 45°. The spectrometer was calibrated to the copper and gold reference lines (932.62 eV and 83.96 eV); the minimum detector resolution measured at the silver peak (Ag3*d*_5/2_) was 0.6 eV with a pass energy of 23.5 eV. The measurements were carried out at room temperature and a base pressure of 2 × 10^−6^ Pa. To avoid charging effects all measurements were performed with charge compensation with a cold cathode electron flood source and low energy argon ions. All spectra (*N* = 12) were shifted to the C1*s* carbon peak (248.6 eV). For data analyses the software MultiPak (version 9.9.0.8, Ulvac-phi, Inc., Chigasaki, Japan) was applied.

To ensure a normal distribution (α = 0.05), a Kolmogorov–Smirnov normality test was used. The data were analysed with a Tukey honestly significant difference test (α = 0.05) to control the results for statistical differences between the variants.

## 3. Results and Discussion

**Figure 2:**
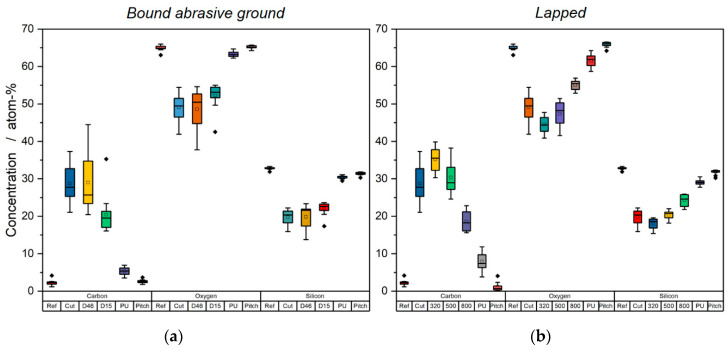
Carbon, oxygen and silicon concentration after each manufacturing step for bound abrasive ground (**a**) and lapped (**b**) samples. For explanation of the particular sample designations given in the x-axis, see Figure 1.

The chemical composition of the investigated sample surfaces after each manufacturing step is shown in Figure 2. Here, merely the glass-forming elements oxygen and silicon as well as the major contaminant of interest, carbon, are considered. It can be seen that the carbon content, which was almost zero in the case of the reference sample, i.e., the fused silica bulk material, rapidly increased due to cutting. After a slight further increase in the course of pre-grinding and rough lapping, the carbon concentration successively decreased and approached or even reached the initially measured reference value. This also applied to the oxygen and silicon content and its ratio, i.e., the nominal stoichiometry of the investigated glass, O/Si = 66%/33% = 2. This value can only be observed after polishing. In the course of cutting, grinding and lapping, the glass surface is thus covered by a carbonaceous layer, which is successively removed by each subsequent manufacturing step.

The origin of surface-adherent carbon or carbonaceous compounds was identified quite easily: As mentioned in Section 2.1, diamond (and thus pure carbon) grains were used for cutting and bound abrasive grinding and silicon carbide grains were applied for lapping. A certain rubbed-off fraction of these operating materials was transferred to the samples as a result of fretting or rubbing wear between the used tools and the glass surface [21]. Moreover, water was used for each manufacturing step. For cutting and grinding, it was mixed with a cooling lubricant made of mineral oil—and thus basically consisting of hydrocarbons. For lapping, it was used for mixing the lapping suspension. Since water contains a certain amount of (partially dissolved) carbon [2,3] it represented a further source for carbon contamination. Finally, both polishing pad materials—polyurethane and pitch—were composed of hydrocarbons and carbonaceous compounds, explaining the residual carbon contamination after polishing.

An interesting aspect was observed when comparing the finished polished surfaces. It turns out that the lowest carbon content and thus the highest surface purity or cleanliness (not to be confused with “surface cleanliness” as specified by code number 5 in ISO 10110) was achieved when using pitch as polishing pad material. This observation or effect can be explained by the basic differences in the particular pad material–glass interactions as discussed by various authors in the past [22,23,24,25,26,27].

The measured curves of bound abrasive ground and lapped samples showed a similar qualitative behaviour. This implies that the contamination by carbon can be attributed to the same basic underlying mechanism. The slight differences in the absolute values can be explained by the different tools and operating materials and the differences in tool–surface interactions during bound abrasive grinding on the one hand and lapping on the other hand.

**Figure 3:**
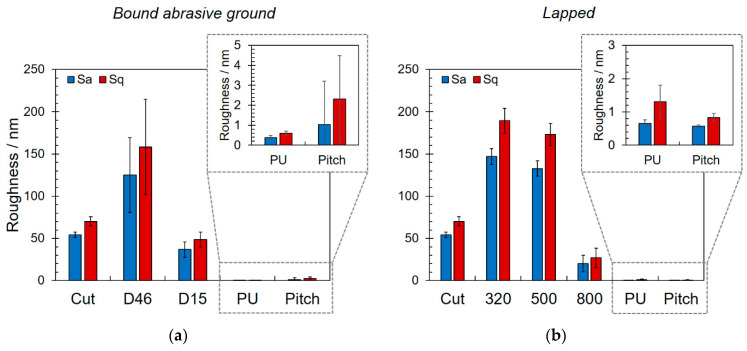
Average area roughness Sa and root mean square area roughness after each manufacturing step for bound abrasive ground (**a**) and lapped (**b**) samples. For explanation of the particular sample designations given in the x-axis, see Figure 1.

As shown in Figure 3, the observed carbon concentration distribution correlated quite well with surface roughness as determined via AFM measurements. As expected, surface roughness decreased continuously in the course of grinding and lapping. It can further be seen that the cut surface had a lower roughness than the pre-ground or rough and medium lapped. This can be explained by the significant difference in tool–surface interaction during cutting on the one hand and grinding or lapping on the other hand. In the first case, abrasion was induced along a predominant direction due to the rotation and thus direction of primary motion of the cut-off wheel. According to DIN 4760 and 4761, macroscopic surface roughness can be classified into two main categories: (i) roughness in the form of grooves or rills, which is the 3rd-order form deviation, and (ii) roughness in the form of striations or cones, i.e., the 4th-order form deviation [28]. Cut surfaces preferentially feature a 3rd-order roughness, whereas ground or lapped surfaces show a 4th-order-like topography.

The direct correlation of the carbon contamination and surface roughness can be attributed to the particular surface area available for the adsorption of hydrocarbons and carbonaceous contaminants where the interaction increases with increasing roughness [29]. Moreover, a higher roughness comes along with an increased number and depth of micro cracks and subsurface damages. As a rule, the micro crack depth at glass surfaces can be estimated to amount to one-third of the equivalent diameter of the grit size of the used grains [1]. In addition, the depth of subsurface damage is directly related to surface roughness; it is given by the product of the peak-to-valley roughness and a glass-dependent constant [30]. The higher the roughness, the higher the number and volume of the abovementioned defects that act as capillaries for the accumulation of contaminants.

The differences in residual carbonic contaminants after polishing can be attributed to the different coefficients of friction of fused silica and polyurethane (0.622 [22]) on the one hand and pitch (0.735 [22]) on the other hand.

## 4. Conclusions

It can be summarised that each classical optics manufacturing step leads to a certain carbon or carbonaceous contamination of the glass surface, where the highest amounts of impurities occur during prefabrication. This includes cutting, pre-grinding and rough or medium lapping, where higher surface roughness results. The observed contaminations cover the glass surface and most likely originate from wear debris of the used tools and residues of the operating materials. The extent of contamination decreases in the course of manufacturing and with decreasing surface roughness or effective surface, respectively. This observation is of interest for further processing of ground glass surfaces and especially for gluing or cementing where adhesion failure may be attributed to carbonic surface-adherent contaminants.

The lowest carbon contamination is found in case of lapped and pitch polished surfaces. This approach is thus the preferable one for the realisation of optical components with high surface purity.

Due to the multiplicity of actuating variables, a definite description of the glass polishing processes is highly complex and does not represent the focus of the present work. However, the observed behaviour and reported results may contribute to a better understanding of glass polishing processes. For this purpose, further investigations will be performed in ongoing work. This includes FTIR- and depth-resolved XPS-measurements as well as the analytical decomposition of relevant carbon peaks in order to identify alterations in chemical bonds in the course of manufacturing.

## Data Availability

The data presented in this study are available on request from the corresponding author.
